# Electromyographic Evaluation of the Impacts of Different Insoles in the Activity Patterns of the Lower Limb Muscles during Sport Motorcycling: A Cross-Over Trial

**DOI:** 10.3390/s19102249

**Published:** 2019-05-15

**Authors:** Israel Casado-Hernández, Ricardo Becerro-de-Bengoa-Vallejo, Marta Elena Losa-Iglesias, Daniel López-López, David Rodríguez-Sanz, Eva María Martínez-Jiménez, César Calvo-Lobo

**Affiliations:** 1 Facultad de Enfermería, Fisioterapia y Podología, Universidad Complutense de Madrid, 28040 Madrid, Spain; israelcasado@yahoo.es (I.C.-H.); ribebeva@ucm.es (R.B.-d.-B.-V.); davidrodriguezsanz@gmail.com (D.R.-S.); eva.hache2@hotmail.com (E.M.M.-J.); 2 Faculty of Health Sciences, Universidad Rey Juan Carlos, 28922 Alcorcón, Spain; marta.losa@urjc.es; 3 Research, Health and Podiatry Unit, Department of Health Sciences, Faculty of Nursing and Podiatry, Universidade da Coruña, 15403 Ferrol, Spain; 4 Nursing and Physical Therapy Department, Institute of Biomedicine (IBIOMED), Faculty of Health Sciences, Universidad de León, 24401 Ponferrada, Spain; ccall@unileon.es

**Keywords:** foot insoles, electromyography, joint instability, muscle contractions, motorcycling, plantar pressure

## Abstract

Customized foot insoles (CFI) have been recognized to reduce the prevalence of foot disorders in sport. The aim of this study was to evaluate the effect of four types of CFI on the activity patterns of the lower limb muscles (LLM) in healthy people during sport motorcycling. Methods: This was a cross-over trial (NCT03734133. Participants were recruited from an outpatient foot specialist clinic. Their mean age was 33 ± 5.14 years. While participants were sport motorcycling in a simulator, the electromyography (EMG) function was registered for LLM via surface electrodes. Participants completed separate tests while wearing one of four types of CFI: (1) only polypropylene (58° Shore D), (2) selective aluminum (60 HB Brinell hardness) in metatarsal and first hallux areas and polypropylene elsewhere (58° Shore D), (3) ethylene vinyl acetate (EVA) (52° Shore A), and (4) standard EVA (25° Shore A) as the control. Results: The activity patterns of the LLM while sport motorcycling showed significantly lower peak amplitude for the selective aluminum CFI than the other types of CFI. Conclusion: EMG amplitude peaks for several LLM were smaller for the hardest CFI (selective aluminum 60 HB Brinell hardness) than the other CFIs (polypropylene 58° Shore D, EVA 52° Shore A, and standard EVA 25° Shore A), except for the fibularis longus in right curves that is increased when the knee touches the road increasing the stability.

## 1. Introduction

Motorcycle racers ride on speed tracks with multiples curves, moving their bodies by taking different postures as they attempt to minimize their lap times. Motorcycle riders take aerodynamic positions, flexing their hips and knees, and also changing to a braking posture. In different phases of cornering, a rider shifts his mass around the center of mass of the motorcycle from one footpeg to another footpeg. Leaning the motorcycle into corners improves the rider’s performance. To achieve this, centrifugal forces have to be balanced and the rider must make rapid changes of lateral, medial, and anteroposterior movements [1].

Lower limb muscles play a relevant role in motorcycling performance through the forces produced by the feet and footpeg. Studies show the importance of motorcycle riders being in good physical shape to reduce muscle fatigue from the large loads to which they are exposed and thereby improve their performance [2]. Thus, motorcycle riders show an increase in the reactive forces of the joints during riding and the associated activity patterns of the LLM related to footwear and the footpeg. These forces and activity patterns may produce various musculoskeletal conditions, such as (1) Achilles tendinopathy, (2) alterations in plantar pressures, (3) muscle strains, (4) knee pain, (5) ligament sprains, (6) metatarsalgia, (7) patellofemoral syndrome, (8) plantar fasciitis, (9) tendon injuries, and (10) paresthesia [3,4,5,6].

These injuries suggest a variety of potential causes: (1) abnormality of LLM, (2) biomechanical dysfunction, (3) characteristics of the riding environment, (4) male gender, (5) type of boot or shoes, and (6) foot pedal force. These factors are complex and related, and their precise roles in causing injury are not clear [7,8,9,10,11,12].

No prior investigation has evaluated electromyography (EMG) activity patterns of LLM or the effects of customized foot insoles (CFI) on such patterns during sport motorcycling. Previous reports have recorded significant variations in the EMG activity patterns of LLM during cycling [13,14,15].

In this study, we evaluated the effects of four types of CFI in the activity patterns of the LLM in healthy people during sport motorcycling. We hypothesized that participants wearing different types of CFI might vary in their EMG function patterns of the LLM. Our main goal was to determine which material of CFI limits the EMG patterns the most.

## 2. Materials and Methods

### 2.1. Design and Sample

Participants were nine healthy motorcycle riders recruited from an outpatient foot specialist clinic in Madrid (Spain) from November to December 2018 using a non-random consecutive sampling method. The inclusion criteria included the following: (1) at least eighteen years old, (2) no medical problems in the clinical record nor a family history with relevant illnesses, and (3) written informed consent to participate in our research. The exclusion criteria were as follows: (1) medical trauma or a history of leg problems, (2) musculoskeletal disturbances, (3) vascular disease, (4) refusal to give written informed consent, and (5) inability to understand the protocol to participate in our research.

We conducted a cross-over trial study (prospectively registered in ClinicalTrials.gov as NCT03734133). Our report follows the guidelines and checklist in the Template for Intervention Description and Replication (TIDieR) [16].

### 2.2. Procedure

The baseline assessment comprised an interview that focused on (1) overall medical health, (2) anthropometric and socio-demographic characteristics (age, sex, height, weight, BMI, and Spanish foot size), (3) chronic disorders and illnesses (e.g., arthritis, diabetes mellitus, and musculoskeletal disturbances), and (4) recreational activities. A senior podiatrist also assessed each rider with a complete physical clinical evaluation and measured anthropometric variables such as height, weight, and body mass index.

Before testing commenced, the participants were given a two-minute acclimation period to drive on the motorcycle simulator. During this period, participants drove on the motorcycle simulator to adjust to the surroundings and ensure the shoes were comfortable. Each motorider wore their own shoes with a flat thin rubber sole.

In the simulator CKU28^®^ (Mecanitzats Muntada SL, Manlleu, Spain), the riders drove the Circuit of the Americas race (Austin, TX, USA) in which professional sport motorcyclists compete [17]. During the simulation, riders drove the race course, making the same movements as real circuit motorcycle riders would need to make. Riders had to accelerate to maximum speed and then, when taking a curve, decelerate. They needed to raise and lower their bodies on the motorcycle simulator to the degrees needed to complete the course. The simulator had a maximum inclination of 60° and riders rested their knees on the ground just as they do on real motorcycles. Riders completed the race at approximately 250 km/h in straightaways and at decreased speeds in curves over a period of 20 min of Racing for each CFI test.

We evaluated four types of CFI: (1) only polypropylene (58° Shore D), (2) selective aluminum (60 HB Brinell hardness) in the metatarsal and first hallux areas and polypropylene elsewhere (58° Shore D), (3) ethylene vinyl acetate (EVA) (52° Shore A), and (4) standard EVA (25° Shore A) as control. We tested each rider with each type of CFI and the order of CFIs was randomly assigned using a random number table. The order of CFIs was the same across all riders.

Before testing, each motorcyclist was situated in a calm place to fix surface electrodes (positive, negative, and earth interfaces) with a built-in preamplifier on the skin over six muscle areas on the right leg: (1) fibularis brevis, (2) fibularis longus, (3) tibialis anterior, (4) gastrocnemius lateralis, (5) gastrocnemius medialis, and (6) soleus. The belly of each muscle was marked on the skin with a permanent marker, swabbed with a 70% alcohol solution and then shaved. The skin was then abraded with skin sandpaper to reduce the electrical impedance to less than 5 kX. All active electrodes were placed 2 cm apart, parallel with the alignment of the muscle fibers, and secured with hypoallergenic tape. A stretch “underwrap” bandage was placed over the electrodes to prevent electrode movement [18].

The precise locations of the EMG electrodes were as follows:

- Fibularis brevis: The electrode was placed anterior to the tendon of the muscle peroneus longus at 25% of the line beginning from the tip of the lateral malleolus to the fibula head, and oriented along this line.
- Fibularis longus: The electrode was placed at 25% of the line beginning from the tip of the head of the fibula to the tip of the lateral malleolus, and oriented along this line.
- Tibialis anterior: The electrode was placed at 1/3 of the line beginning from the tip of the fibula to the tip of the medial malleolus, and oriented along this line. We placed the tibialis anterior electrodes carefully in line and parallel to the fibula because cross-talk can exist between the tibialis anterior and peroneus longus, particularly when the peroneus longus electrodes are placed too anteriorly on the leg. Manual muscle testing was performed for each muscle area to confirm correct placement.
- Gastrocnemius lateralis: The electrode was placed at 1/3 of the line beginning from the head of the fibula to the heel, and oriented along this line.
- Gastrocnemius medialis: The electrode was placed on the most prominent bulge of the muscle and oriented along the length of the leg.
- Soleus: The electrode was placed at 2/3 of the line beginning from the medial condyle of the femur to the medial malleolus, and oriented along this line.

For normalization purposes, before performing each trial, the participant was instructed on how to perform a maximum voluntary isometric contraction (MVIC) for each muscle based on a technique described by Yang and Winter [19]. Three MVICs were recorded for each muscle within a recording time of eight seconds. This allowed time for the participant to build up the contraction and for the tester to stabilize the foot. Thus, the MVIC was measured over a four-second window only. The participants were instructed to exert a maximum effort against the resistance of the tester and were given verbal encouragement while doing so. The rationale for this test is to normalize the maximal amplitude of a submaximal task, such as walking or riding a motorcycle, to the amplitude of the MVIC produced by the participant. One of the known limitations related to the reliability of the MVIC is the participant’s ability to produce an MVIC sincerely [19]. Although the participant’s effort can vary between each contraction, using the MVIC is better than not using a normalization technique at all [20]. To reduce the effect of muscle fatigue between succeeding contractions, participants were given one-minute rest periods [21].

The EMG electrodes were inserted into a lightweight backpack EMG interface unit (model MWX8). The interface unit was wired to the filtering hardware by an overhead pulley system. This was connected to a personal computer where EMG information was recorded using DataLINK (v.5.0) (Biometrics Ltd., Ladysmith, VA, USA).

We measured the signal activity patterns with surface electromyography (EMG) sensors (SX230) (Biometrics Ltd., Ladysmith, VA, USA), using the protocol design of SENIAM to comply with a non-invasive evaluation of muscles [12]. Electrode specifications in this study were as follows: amplification, single differential; inter-electrode distance, 2 cm; contact sensors, two 0.37 × 0.20 × 0.06 cm silver bars; preamplifier gain, 1000; input impedance, >10,000,000 MΩ; and common mode rejection ratio, >96 dB. The main amplifier unit feature was a gain of 1000-fold and frequency response of 20–460 Hz. Surface electromyography data were sampled at 1000 Hz and recorded by a computerized data logger (MWX8 Biometrics Ltd., Ladysmith, VA, USA).

We chose the 1 kHz data sampling rate because raw EMG signals were collected, converted with DataLINK, and recorded prior to producing the filtered/rectified data. This ensured that the peaks and onsets of activity were reproduced with subsequent data, and enabled the verification that movement artifacts were minimal [18]. Electromyography signals recorded during the selected times were filtered with a band pass filter (bandwidth of 20–460 Hz) [21,22]. EMG parameters such as frequency content and waveform shape may require sampling rates more than twice the Nyquist rate [23] for accurate interpretation. However, for the analysis of amplitude and timing parameters, over-sampling is unnecessary [24].

The average rectified value (ARV) was calculated. The total ARV and ARV during riding for each muscle and each participant were calculated and normalized by the peak ARV for each participant. To identify the muscular activation phase, the onset and offset of surface electromyography were assessed. Phases of muscular activation were defined by the period when the signal was above a threshold of 25% of the peak ARV for each muscle and participant [25,26]. The signal activity patterns related to the LLM were registered for each CFI test during the last five minutes of the simulated race.

For each riding, “raw” recordings (without bandpass or RMS/envelope filtering) were taken to compare with filtered recordings. The signals were then filtered during testing for each participant.

We calculated the root mean square (RMS) of each signal for each subject, and used a filter bank of 10 wavelets. We also computed center frequencies that characterize the wavelets [27]. These operations yielded a time series of EMG intensities for each wavelet (wavelet domain). These time series were re-sampled at 200 Hz and divided by the square root of the sum of the intensities of the data points in all wavelet domains. This yielded intensity patterns, which we used in blocks for further analysis.

During the collection and analysis of EMG tracings, the operator was blinded to the condition being evaluated. Muscle onset was determined by visual observation as the time when a rapid and continuous increase in voltage occurred that was unrelated to movement artifacts or noise interference.

Lastly, all EMG electrodes were placed on the muscles of the right lower extremity of each motorider, so the right curve means that the right lower extremity is touching the floor with the knee and applying force against the footpeg, while the left curve means that the same lower limb must move towards the motorcycle body part to maintain stability.

### 2.3. Ethical Considerations

The study protocol was reviewed and approved by the commission of the Clinical Research Ethics Committee of Hospital Clínico San Carlos, Madrid, Spain (number C.I. 18/506-E). All riders gave written informed consent to participate in our research. Furthermore, our study procedures complied with the ethical principles and human research guidelines of the World Medical Association in the Declaration of Helsinki.

#### Sample Size Calculation

We calculated the sample size necessary for our research goals with a program developed by the Clinical Epidemiology and Biostatistics Unit of the Universidade da Coruña (A Coruña, Spain) (http://www.fisterra.com/mbe/investiga/9muestras/9muestras2.asp) [28]. We based our calculations on prior results obtained by McCulloch on the effect of pedal type on EMG activity patterns in muscle recruitment (16.9 ± 7.2% for a lateral translation pedal and 30.9 ± 8.5% for a standard pedal) [29]. With these results, a sample of 8 riders would give 80% analysis power (beta = 20%), to detect a significant effect (alpha of 0.05) in a two-tailed test for this cross-over experimental study.

### 2.4. Statistical Analysis

We assessed all variables for normality with the Shapiro–Wilk test and considered a variable to have a normal distribution if the probability value for a test was greater than or equal to 0.05. To describe riders’ demographic characteristics, we computed the means, standard deviations, and 95% confidence intervals of rider age, BMI, Spanish foot size, height, and weight.

We measured intra-trial reliability with the three records for each CFI for each rider. To evaluate reliability within trials in each rider, we computed intra-class correlation coefficients. For interpreting ICC values, we considered values less than 0.40 as poor, values between 0.40 and 0.59 as fair, values between 0.60 and 0.74 as good, and values 0.75 or greater as excellent [30,31]. Portney and Watkins [32] proposed that reliability coefficients greater than 0.90 were sufficient for clinical measurement. We also calculated the mean scores and the standard error of measurement (SEM) [30]. We used Brand and Altman’s [33] formula for the SEM as follow: SEM = SD × sqrt (1 − ICC).

The minimal detectable change (MDC) can be used to assess the minimal magnitude of change required to be 95% confident that the observed change between the 2 tests reflects the true change and not measurement error [34]. The MDC was calculated as: 1.96 × SEM × √2.

For demonstrating the effect size of the comparisons, the Cohen d coefficient was calculated. Cohen’s d effect size can be interpreted as follows: values ≤0.20 indicate slight effects, values between 0.20 and 0.49 indicate fair effects, values between 0.50 and 0.79 indicate moderate effects, and values larger than 0.79 indicate large effects [35].

We computed nonparametric Mann–Whitney U tests to assess differences among CFIs groups. For all tests, we considered *p* values < 0.05 to be statistically significant. We used SPSS 19.0 for Windows (IBM, Chicago, IL, USA) to perform all analyses.

## 3. Results

### 3.1. Characteristics of the Riders

Table 1 shows the anthropometric and demographic characteristics of the nine healthy riders who participated in the study.

### 3.2. Normality of EMG Variables of the LLM by CFI

Table 2 shows the Shapiro–Wilk test results on the normality of the EMG variables of the LLM by CFI types for left and right curves in the simulator.

#### Intraclass Correlation Coefficient for EMG in LLM

Table 3 shows the ICC values for EMG activity in the LLM by CFI types for left and right turns in the simulator.

Table 4 shows the EMG activity patterns of the LLM by type of CFI for left and right curves in the simulator. Table 5 shows comparisons between pairs of CFIs on these activity patterns.

The selective aluminum (60 HB Brinell hardness) CFI generated statistically significant smaller amplitude peaks than Eva 52% and Polypropylene 58% CFIs compared with Standard EVA 25% CFI for left and right curves in the simulator (*p* < 0.001) in all muscles studied except for the fibularis longus right curve that is increased (*p* < 0.001).

In another hand, polypropylene 58° CFI produced statistically significant smaller amplitude peaks than Eva 52% CFI compared with Standard EVA 25% for left and right curves in the simulator for all muscles (*p* < 0.001) except also, for the fibularis longus right curve which increased (*p* < 0.001).

The results using Eva 52% are lower compared with Standard EVA 25% for left and right curves in the simulator for all muscles (*p* < 0.001) except for the fibularis longus right curve which increased (*p* < 0.001).

## 4. Discussion

Researchers have carried out several investigations on EMG activity patterns of LLM during cycling [36,37,38]. Notably, Bousie et al. [36] found that contoured insoles increased the contact area of the foot in stationary cycling. In addition, Casado et al. reported variations in motorcycle riders’ plantar pressures with different CFIs when riding in a motorcycle simulator, concluding that metatarsal and hallux pressures decreased with the hardest CFI [17].

Riding on a speed circuit with multiples curves and bends requires precise control of the dynamics of the supersport motorcycle. Stability on the motorcycle is achieved with the hands on the handlebars, buttocks on the seat, legs, and feet on the footpegs. Motorcycle riders perform load transfers during changes in speed, lateral tilts on curves, and changes in direction, demanding repeated physical effort [39,40].

To our knowledge, our investigation is the first to use EMG for recording the muscular activity of the LLM in motorcycling with different CFI. We evaluated the effects of four types of CFI: (1) only polypropylene (58° Shore D), (2) selective aluminum (60 HB Brinell hardness) in the metatarsal and first hallux areas and polypropylene elsewhere (58° Shore D), (3) ethylene vinyl acetate (EVA) (52° Shore A), and (4) standard EVA (25° Shore A; used as control).

In our research, we studied the variation of EMG activity peak amplitudes in the right lower leg of each rider taking right and left curves as they used different CFIs. To maintain the stability of the motorcycle when taking a curve, a rider touches the road on the inner part of the curve with his knee and applies force against the footpeg with the plantar aspect of his first metatarsal bone. When taking the curve, the rider also moves his other leg toward the body of the motorcycle. The fibularis longus is the main muscle that keeps the first metatarsal bone in plantarflexion against the footpeg and therefore is one of the most important muscles for maintaining stability as the knee touches the road during the curve.

The select aluminum CFI had significantly smaller amplitude peaks than the other CFIs for both left and right curves in the simulator for the tibialis anterior, gastrocnemius lateralis, gastrocnemius medialis, soleus, and fibularis brevis. For the fibularis longus, the select aluminum CFI had significantly smaller amplitude peaks than other CFIs in left curves, but significantly higher amplitude peaks than other CFIs in right curves. We suspect that the high activity of the fibularis longus in right curves, when the knee touches the road, increases stability while riding. Also, decreased activity of the other LLM in left and right curves would likely contribute to decreased fatigue when riding.

More studies are needed on muscular activity during sport motorcycling because there are currently speed circuits with a greater number of right and left curves than in the circuit our riders rode. The data we obtained would help estimate the overloading of LLM in such contexts.

A limitation of our study is the lack of inertial forces and steering control challenges in our simulator that occur when riding a motorcycle on a real speed circuit. Further research is needed on the physical effects of motorcycling during actual sport races.

## 5. Conclusions

EMG amplitude peaks for several LLM were smaller for the hardest CFI (selective aluminum 60 HB Brinell hardness) than the other CFIs (polypropylene 58° Shore D, EVA 52° Shore A, and standard EVA 25° Shore A), except for the fibularis longus in right curves that is increased when the knee touches the road increasing the stability.

## Figures and Tables

**Table 1:** Anthropometric and demographic characteristics of the sample of riders.

Variables	Mean ± SD (n = 9)	Range (Min–Max) (n = 9)	95% CI (n = 9)
Age (years)	33 ± 5.14	26–40	29.04–36.96
Height (cm)	175 ± 44	164–190	169.77–181.12
Weight (Kg)	71 ± 44	63–78	67.50–75.38
BMI (Kg/m^2^)	23.45 ± 1.04	22.22–24.82	22.65–24.26
Spanish foot size	42.22 ± 2.86	37–47	40.02–44.42
Abbreviations: SD: standard deviation; Min: minimum; Max: maximum; 95% CI: 95% confidence interval; cm: centimeters; Kg: kilograms; Kg/m^2^: kilograms/meter^2^			

**Table 2:** Normality of the electromyography variables of the lower limb muscles for particular types of customized foot insoles for left and right curves in the simulator.

Variable LLM and Curve	*p*-Value	Variable LLM and Curve	*p*-Value
Tibialis Anterior standard EVA left curve	0.232	Soleus standard EVA left curve	0.628
Tibialis Anterior standard EVA right curve	0.934	Soleus standard EVA right curve	0.791
Tibialis Anterior EVA left curve	0.080	Soleus EVA left curve	0.840
Tibialis Anterior EVA right curve	0.086	Soleus EVA right curve	0.507
Tibialis Anterior polypropylene left curve	0.656	Soleus polypropylene left curve	0.863
Tibialis Anterior polypropylene right curve	0.016	Soleus polypropylene right curve	0.375
Tibialis Anterior selective aluminum left curve	0.115	Soleus selective aluminum left curve	0.787
Tibialis Anterior selective aluminum right curve	0.006	Soleus selective aluminum right curve	0.042
Gastrocnemius lateralis standard EVA left curve	0.319	Fibularis brevis standard EVA left curve	0.972
Gastrocnemius lateral standard EVA right curve	0.716	Fibularis brevis standard EVA right curve	0.264
Gastrocnemius lateralis EVA left curve	0.636	Fibularis brevis EVA left curve	0.084
Gastrocnemius lateralis EVA right curve	0.095	Fibularis brevis EVA right curve	0.495
Gastrocnemius lateralis polypropylene left curve	0.579	Fibularis brevis polypropylene left curve	0.059
Gastrocnemius lateralis polypropylene right curve	0.089	Fibularis brevis polypropylene right curve	0.753
Gastrocnemius lateralis selective aluminum left curve	0.338	Fibularis brevis selective aluminum left curve	0.081
Gastrocnemius lateralis selective aluminum right curve	0.769	Fibularis brevis selective aluminum right curve	0.852
Gastrocnemius medialis standard EVA left curve	0.810	Fibularis longus standard EVA left curve	0.468
Gastrocnemius medialis standard EVA right curve	0.019	Fibularis longus standard EVA right curve	0.460
Gastrocnemius medialis EVA left curve	0.250	Fibularis longus EVA left curve	0.017
Gastrocnemius medialis EVA right curve	0.233	Fibularis longus EVA right curve	0.799
Gastrocnemius medialis polypropylene left curve	0.644	Fibularis longus polypropylene left curve	0.144
Gastrocnemius medialis polypropylene right curve	0.812	Fibularis longus polypropylene right curve	0.728
Gastrocnemius medialis selective aluminum left curve	0.084	Fibularis longus selective aluminum left curve	0.276
Gastrocnemius medialis selective aluminum right curve	0.810	Fibularis longus selective aluminum right curve	0.291
Abbreviations: EVA, ethylene vinyl acetate; Right curve, Knee touching the road; Left curve, Lower extremity towards the motorcycle body part. *p* Values are from Shapiro-Wilk tests			

**Table 3:** Intraclass correlation coefficients, standard error of measurement and minimal detectable change for electromyography (EMG) activity patterns of the lower limb muscles by types of customized foot insoles for left and right curves in the simulator.

**Variable LLM and Curve**	**ICC (95% CI)**	**SEM**	**MDC**	***p*-Value**
Tibial Anterior standard EVA left curve	0.980 (0.940–0.995)	0.499	1.384	<0.001
Tibialis Anterior standard EVA right curve	0.835 (0.481–0.960)	0.995	2.759	0.002
Tibialis Anterior EVA left curve	0.942 (0.824–0.986)	0.352	0.975	<0.001
Tibial Anterior EVA curve right	0.785 (0.295–0.948)	1.433	3.971	0.006
Tibialis Anterior polypropylene left curve	0.935 (0.803–0.984)	1.050	2.912	<0.001
Tibialis Anterior polypropylene right curve	0.981 (0.940–0.995)	0.416	1.154	<0.001
Tibialis Anterior selective aluminum left curve	0.969 (0.904–0.992)	0.868	2.406	<0.001
Tibialis Anterior selective aluminum right curve	0.933 (0.792–0.983)	0.774	2.145	<0.001
Gastrocnemius lateralis standard EVA left curve	0.932 (0.797–0.983)	0.386	1.070	<0.001
Gastrocnemius lateral standard EVA right curve	0.995 (0.866–0.989)	0.330	0.915	<0.001
Gastrocnemius lateralis EVA left curve	0.974 (0.916–0.994)	0.221	0.612	<0.001
Gastrocnemius lateralis EVA right curve	0.956 (0.868–0.989)	0.501	1.390	<0.001
Gastrocnemius lateralis polypropylene left curve	0.950 (0.707–0.989)	0.101	0.279	<0.001
Gastrocnemius lateralis polypropylene right curve	0.974 (0.917–0.994)	0.585	1.622	<0.001
Gastrocnemius lateralis selective aluminum left curve	0.618 (−0.213–0.907)	0.655	1.816	0.053
Gastrocnemius lateralis selective aluminum right curve	0.986 (0.959–0.997)	0.380	1.053	<0.001
Gastrocnemius medialis standard EVA left curve	0.972 (0.913–0.993)	0.321	0.891	<0.001
Gastrocnemius medialis standard EVA right curve	0.980 (0.939–0.995)	0.856	2.372	<0.001
Gastrocnemius medialis EVA left curve	0.506 (−0.412–0.875)	2.488	6.897	0.101
Gastrocnemius medialis EVA right curve	0.995 (0.984–0.999)	0.324	0.898	<0.001
Gastrocnemius medialis polypropylene left curve	0.868 (0.604–0.967)	0.516	1.430	<0.001
Gastrocnemius medialis polypropylene right curve	0.985 (0.955–0.996)	0.311	0.862	<0.001
Gastrocnemius medialis selective aluminum left curve	0.994 (0.981–0.998)	0.225	0.625	<0.001
Gastrocnemius medialis selective aluminum right curve	0.980 (0.935–0.995)	0.296	0.819	<0.001
Soleus standard EVA left curve	0.981 (0.942–0.995)	1.083	3.003	<0.001
Soleus standard EVA right curve	0.943 (0.829–0.986)	2.151	5.963	<0.001
Soleus EVA left curve	0.823 (0.474–0.956)	3.315	9.189	0.001
Soleus EVA right curve	0.989 (0.968–0.997)	0.915	2.535	0.001
Soleus polypropylene left curve	0.939 (0.815–0.985)	0.620	1.718	<0.001
Soleus polypropylene right curve	0.849 (0.531–0.963)	2.304	6.387	<0.001
Soleus selective aluminum left curve	0.972 (0.917–0.993)	0.540	1.498	<0.001
Soleus selective aluminum right curve	0.950 (0.836–0.988)	1.346	3.731	0.001
**Variable LLM and curve**	**ICC (95% CI)**	**SEM**	**MDC**	***p*-Value**
Fibularis brevis standard EVA left curve	0.801 (0.421–0.950)	2.360	6.541	<0.001
Fibularis brevis standard EVA right curve	0.862 (0.587–0.966)	1.612	4.469	<0.001
Fibularis brevis EVA left curve	0.951 (0.850–0.988)	1.260	3.491	<0.001
Fibularis brevis EVA right curve	0.988 (0.962–0.997)	0.620	1.719	<0.001
Fibularis brevis polypropylene left curve	0.950 (0.851–0.988)	0.762	2.114	<0.001
Fibularis brevis polypropylene right curve	0.956 (0.864–0.989)	0.990	2.744	<0.001
Fibularis brevis selective aluminum left curve	0.981 (0.941–0.995)	0.804	2.227	<0.001
Fibularis brevis selective aluminum right curve	0.950 (0.843–0.988)	0.892	2.473	<0.001
Fibularis longus standard EVA left curve	0.202 (−0.892–0.780)	3.716	10.301	0.308
Fibularis longus standard EVA right curve	0.888 (0.654–0.972)	2.349	6.512	<0.001
Fibularis longus EVA left curve	0.992 (0.975–0.998)	1.042	2.888	<0.001
Fibularis longus EVA right curve	0.965 (0.892–0.991)	1.478	4.097	<0.001
Fibularis longus polypropylene left curve	0.996 (0.989–0.999)	0.844	2.339	<0.001
Fibularis longus polypropylene right curve	0.990 (0.971–0.998)	0.934	2.589	<0.001
Fibularis longus selective aluminum left curve	0.974 (0.921–0.994)	2.628	7.285	<0.001
Fibularis longus selective aluminum right curve	0.989 (0.967–0.997)	0.824	2.285	<0.001
Abbreviations: 95% CI, 95% confidence interval; ICC, intraclass correlation coefficient; SEM: standard error of the mean; MDC: minimal detectable change; EVA, ethylene vinyl acetate; Right curve, Knee touching the road; Left curve, Lower extremity towards the motorcycle body part				

**Table 4:** Maximum muscular activity of the lower limb muscles (LLM) as measured by EMG for different customized foot insoles (CFI) for left and right curves in the simulator.

Variables	Standard EVA 25% (25° Shore A)		EVA 52% (52° Shore A)		Polypropylene 58% (58° Shore D)		Selective Aluminum 60 HD (60 HB Brinell Hardness)	
LLM and Curve	Mean ± SD (95% CI)	Median (95% CI)	Mean ± SD (95% CI)	Median (95% CI)	Mean ± SD (95% CI)	Median (95% CI)	Mean ± SD (95% CI)	Median (95% CI)
Tibialis anterior left curve	40.65 ± 2.45	41.36	28.45 ± 3.09	27.57	24.88 ± 3.02	22.91	25.88 ± 2.99	27.14
	(38.76 to 42.53)	(38.97 to 41.58)	(26.08 to 30.83)	(26.60 to 29.42)	(22.56 to 27.19)	(22.48 to 28.33)	(23.58 to 28.17)	(25.73 to 27.58)
Tibialis anterior right curve	49.39 ± 3.53	48.1	40.20 ± 1.46	40.39	32.48 ± 4.12	30.73	29.70 ± 4.93	29.53
	(46.67 to 52.11)	(47.23 to 52.66)	(39.07 to 41.32)	(40.17 to 41.48)	(29.31 to 35.64)	(30.18 to 34.85)	(25.91 to 33.49)	(24.21 to 34.53)
Gastrocnemius lateralis left curve	95.38 ± 4.67	96.17	58.69 ± 2.39	57.70	43.86 ± 3.63	43.22	27.15 ± 3.21	27.04
	(91.79 to 98.97)	(94.17 to 97.32)	(56.85 to 60.52)	(57.27 to 60.19)	(41.06 to 46.65)	(42.46 to 44.69)	(24.68 to 29.62)	(24.62 to 30.08)
Gastrocnemius lateralis right curve	26.28 ± 1.48	26.87	21.82 ± 1.37	21.78	18.06 ± 0.45	17.99	16.75 ± 1.06	16.81
	(25.14 to 27.41)	(24.79 to 27.52)	(20.77 to 22.88)	(21.00 to 22.58)	(17.71 to 18.41)	(17.85 to 18.15)	(15.93 to 17.56)	(16.58 to 17.37)
Gastrocnemius medialis left curve	40.43 ± 6.05	36.56	37.39 ± 4.58	35.96	26.21 ± 2.54	26.83	17.32 ± 2.09	17.31
	(35.78 to 45.09)	(36.00 to 46.15)	(33.87 to 40.91)	(34.92 to 42.17)	(24.26 to 28.16)	(25.06 to 27.10)	(15.71 to 18.93)	(15.64 to 18.49)
Gastrocnemius medialis right curve	37.73 ± 1.92	37.56	31.87 ± 3.54	32.28	19.40 ± 1.42	19.71	8.49 ± 2.91	6.91
	(36.25 to 39.20)	(37.12 to 39.12)	(29.15 to 34.59)	(31.56 to 33.88)	(18.32 to 20.49)	(18.74 to 19.93)	(6.25 to 10.73)	(6.39 to 9.54)
Soleus left curve	77,61 ± 9,01	76.19	63.54 ± 8.72	65.08	55.28 ± 5.93	53.98	51.84 ± 6.02	54.36
	(70.69 to 84,54)	(69.60 to 84.13)	(56.84 to 70.24)	(58.24 to 67.94)	(50.73 to 59.84)	(52.29 to 61.41)	(47.21 to 56.47)	(49.14 to 56.47)
Soleus right curve	58.05 ± 7.86	60.58	57.60 ± 7.88	57.06	39.07 ± 2.51	39.77	33.65 ± 3.23	33.33
	(52.00 to 64.09)	(51.04 to 63.49)	(51.54 to 63.65)	(53.14 to 62.54)	(37.13 to 41.00)	(37.65 to 40.27)	(31.17 to 36.13)	(31.43 to 34.97)
Fibularis brevis left curve	75.13 ± 5.29	75.33	66.97 ± 5.69	68.31	54.87 ± 3.41	56.20	57.78 ± 5.83	56.17
	(71.06 to 79.20)	(71.59 to 78.36)	(62.59 to 71.35)	(60.52 to 71.56)	(52.25 to 57.49)	(53.62 to 57.31)	(53.29 to 62.26)	(54.55 to 57.89)
Fibularis brevis right curve	51.19 ± 4.34	50.00	48.31 ± 5.66	47.76	31.52 ± 4.72	31.34	3.82 ± 3.99	31.72
	(47.85 to 54.53)	(47.51 to 52.95)	(43.96 to 52.67)	(44.44 to 51.80)	(27.90 to 35.15)	(27.96 to 35.53)	(28.75 to 34.88)	(30.93 to 33.28)
Fibularis longus left curve	62.47 ± 7.02	60.77	52.26 ± 7.90	51.61	52.00 ± 9.34	50.29	43.88 ± 7.86	45.18
	(57.07 to 67.87)	(58.68 to 68.92)	(46.19 to 58.34)	(45.63 to 55.96)	(44.82 to 59.18)	(45.77 to 55.54)	(37.83 to 49.92)	(37.20 to 49.91)
Fibularis longus right curve	40.75 ± 4.16	41.67	54.01 ± 11.65	49.00	59.88 ± 13.34	63.85	75.64 ± 16,30	76.86
	(37.54 to 43.95)	(38.35 to 44.05)	(45.06 to 62.96)	(45.34 to 67.09)	(49.63 to 70.14)	(47.19 to 69.52)	(63.11 to 88.18)	(62.52 to 89.20)
Abbreviations: SD: standard deviation; 95% CI: 95% confidence interval; EVA, ethylene vinyl acetate; Right curve, Knee touching the road; Left curve, Lower extremity towards the motorcycle body part								

**Table 5:** Comparisons between types of customized foot insoles in terms of EMG activity of the lower limb muscles for left and right curves in the simulator.

LLM and Curve	EVA 25° Vs EVA 52° *p* Value	EVA 25° Vs Polypropylene 58° *p* Value	EVA 25° Vs Aluminum 60° *p* Value
Tibialis Anterior left curve	<0.001	<0.001	<0.001
Tibialis Anterior right curve	0.001	<0.001	<0.001
Gastrocnemius lateralis left curve	<0.001	<0.001	<0.001
Gastrocnemius lateralis right curve	0.001	<0.001	<0.001
Gastrocnemius medialis left curve	0.194	<0.001	<0.001
Gastrocnemius medialis right curve	0.012	<0.001	<0.001
Soleus left curve	<0.001	<0.001	<0.001
Soleus right curve	0.486	<0.001	<0.001
Fibularis brevis left curve	0.062	0.011	0.001
Fibularis brevis right curve	0.038	0.017	<0.001
Fibularis longus left curve	0.003	0.001	0.001
Fibularis longus right curve	1.000	<0.001	<0.001
Abbreviations: SD: standard deviation; 95% CI: 95% confidence interval; EVA, ethylene vinyl acetate; Right curve, Knee touching the road; Left curve, Lower extremity towards the motorcycle body part			

## References

[1] Ibbot A. (2013). MotoGP Performance Riding Techniques.

[2] Hill E.C., Housh T.J., Smith C.M., Cochrane K.C., Jenkins N.D.M., Cramer J.T., Schmidt R.J., Johnson G.O. (2016). Effect of sex on torque, recovery, EMG, and MMG responses to fatigue. J. Musculoskelet. Neuronal Interact..

[3] Sanner W., O’Halloran W. (2000). The biomechanics, etiology, and treatment of cycling injuries. J. Am. Podiatr. Med. Assoc..

[4] Ramos-Ortega J., Domínguez G., Castillo J.M., Fernández-Seguín L., Munuera P.V. (2014). Angular Position of the Cleat According to Torsional Parameters of the Cyclist’s Lower Limb. Clin. J. Sport Med..

[5] Mellion M.B. (1991). Common Cycling Injuries. Sport Med..

[6] Thompson M.J., Rivara F.P. (2001). Bicycle-related injuries. Am. Fam. Physician.

[7] Horner C.H., O’Brien A.A. (1986). Motorcycle racing injuries on track and road circuits in Ireland. Br. J. Sports Med..

[8] Hinds J.D., Allen G., Morris C.G. (2007). Trauma and motorcyclists; born to be wild, bound to be injured?. Injury.

[9] Aare M., von Holst H. (2003). Injuries from motorcycle and moped crashes in Sweden from 1987 to 1999. Inj. Control Saf. Promot..

[10] Tomida Y., Hirata H., Fukuda A., Tsujii M., Kato K., Fujisawa K., Uchida A. (2005). Injuries in elite motorcycle racing in Japan. Br. J. Sports Med..

[11] Bedolla J., Santelli J., Sabra J., Cabanas J.G., Ziebell C., Olvey S. (2016). Elite motorcycle racing: Crash types and injury patterns in the MotoGP class. Am. J. Emerg. Med..

[12] Varley G.W., Spencer-Jones R., Thomas P., Andrews D., Green A.D., Stevens D.B. (1993). Injury patterns in motorcycle road racers: Experience on the Isle of Man 1989–1991. Injury.

[13] Momeni K., Faghri P.D., Evans M. (2014). Lower-extremity joint kinematics and muscle activations during semi-reclined cycling at different workloads in healthy individuals. J. Neuroeng. Rehabil..

[14] Baum B.S., Li L. (2003). Lower extremity muscle activities during cycling are influenced by load and frequency. J. Electromyogr. Kinesiol..

[15] Hug F., Laplaud D., Lucia A., Grelot L. (2006). EMG Threshold Determination in Eight Lower Limb Muscles During Cycling Exercise: A Pilot Study. Int. J. Sports Med..

[16] Hoffmann T.C., Glasziou P.P., Boutron I., Milne R., Perera R., Moher D., Altman D.G., Barbour V., Macdonald H., Johnston M. (2014). Better reporting of interventions: Template for intervention description and replication (TIDieR) checklist and guide. BMJ.

[17] Casado-Hernández I., Becerro-de-Bengoa-Vallejo R., López-López D., Gómez-Bernal A., Losa-Iglesias M.E. (2018). Aluminum foot insoles reduce plantar forefoot pressure and increase foot comfort for motorcyclists. Prosthet. Orthot. Int..

[18] Hermens H.J., Freriks B., Merletti R., Stegeman D., Blok J., Rau G., Disselhorst-Klug C., Hägg G. European Recommendations for Surface ElectroMyoGraphy Results of the SENIAM Project. https://pdfs.semanticscholar.org/1ab2/8b8afcb1216cab1b2f8da0de246c3d5ed6e8.pdf.

[19] Yang J.F., Winder D.A. (1984). Electromyographic amplitude normalization methods: Improving their sensitivity as diagnostic tools in gait analysis. Arch. Phys. Med. Rehabil..

[20] Knutson L.M., Soderberg G.L., Ballantyne B.T., Clarke W.R. (1994). A study of various normalization procedures for within day electromyographic data. J. Electromyogr. Kinesiol..

[21] Cram J.R., Kasman G.S., Holtz J. (1998). Introduction to Surface Electromyography.

[22] Clancy E.A., Morin E.L., Merletti R. (2002). Sampling, noise-reduction and amplitude estimation issues in Surface electromyography. J. Electromyogr. Kinesiol..

[23] Hunter A.M., St Clair Gibson A., Lambert M., Dennis S., Mullany H., O’Malley M.J., Vaughan C.L., Kay D., Noakes T.D. (2003). EMG amplitude in maximal and submaximal exercise is dependent on signal capture rate. Int. J. Sports Med..

[24] Ives J.C., Wigglesworth J.K. (2003). Sampling rate effects on surface EMG timing and amplitude measures. Clin. Biomech. (Bristol, Avon).

[25] Duc S., Bertucci W., Pernin J.N., Grappe F. (2008). Muscular activity during uphill cycling: Effect of slope, posture, hand grip position and constrained bicycle lateral sways. J. Electromyogr. Kinesiol..

[26] Li L., Caldwell G.E. (1998). Muscle coordination in cycling: Effect of surface incline and posture. J. Appl. Physiol. (1985).

[27] von Tscharner V., Goepfert B. (2003). Gender dependent EMGs of runners resolved by time/frequency and principal pattern analysis. J. Electromyogr. Kinesiol..

[28] Fernández P. (1996). Investigación: Determinación del tamaño muestral Determinación del tamaño muestral. Cad Aten Primaria Actual..

[29] McCulloch R.S. (2018). Influence of lateral pedal translation on muscle recruitment and kinematics in cyclists. J. Exerc. Sci. Fit..

[30] Enderlein G., Fleiss J.L. (2007). The Design and Analysis of Clinical Experiments. Wiley, New York - Chichester - Brislane - Toronto - Singapore 1986, 432 S., £38.35. Biom. J..

[31] Hallgren K.A. (2012). Computing Inter-Rater Reliability for Observational Data: An Overview and Tutorial. Tutor. Quant. Methods Psychol..

[32] Portney L., Watkins M. (2009). Foundations of Clinical Research: Applications to Practice.

[33] Bland J.M., Altman D.G. (1986). Statistical methods for assessing agreement between two methods of clinical measurement. Lancet (Lond. Engl.).

[34] Stratford P.W., Binkley J., Solomon P., Finch E., Gill C., Moreland J. (1996). Defining the minimum level of detectable change for the Roland-Morris questionnaire. Phys. Ther..

[35] Cohen J. (1992). A power primer. Psychol. Bull..

[36] Bousie J.A., Blanch P., McPoil T.G., Vicenzino B. (2013). Contoured in-shoe foot orthoses increase mid-foot plantar contact area when compared with a flat insert during cycling. J. Sci. Med. Sport.

[37] Yeo B.K., Bonanno D.R. (2014). The effect of foot orthoses and in-shoe wedges during cycling: A systematic review. J. Foot Ankle Res..

[38] McCormick C.J., Bonanno D.R., Landorf K.B. (2013). The effect of customised and sham foot orthoses on plantar pressures. J. Foot Ankle Res..

[39] D’Artibale E., Laursen P.B., Cronin J.B. (2017). Profiling the physical load on riders of top-level motorcycle circuit racing. J. Sports Sci..

[40] D’Artibale E., Tessitore A., Capranica L. (2008). Heart rate and blood lactate concentration of male road-race motorcyclists. J Sports Sci..

